# Unveiling efferocytosis-related signatures through the integration of single-cell analysis and machine learning: a predictive framework for prognosis and immunotherapy response in hepatocellular carcinoma

**DOI:** 10.3389/fimmu.2023.1237350

**Published:** 2023-07-27

**Authors:** Tao Liu, Chao Li, Jiantao Zhang, Han Hu, Chenyao Li

**Affiliations:** ^1^Colorectal and Anal Surgery Department, General Surgery Center, First Hospital of Jilin University, Changchun, Jilin, China; ^2^Department of General, Visceral, and Transplant Surgery, Ludwig-Maximilians University, Munich, Germany

**Keywords:** HCC, efferocytosis, single-cell sequencing, immunotherapy, biomarker

## Abstract

**Background:**

Hepatocellular carcinoma (HCC) represents a prominent gastrointestinal malignancy with a grim clinical outlook. In this regard, the discovery of novel early biomarkers holds substantial promise for ameliorating HCC-associated mortality. Efferocytosis, a vital immunological process, assumes a central position in the elimination of apoptotic cells. However, comprehensive investigations exploring the role of efferocytosis-related genes (EFRGs) in HCC are sparse, and their regulatory influence on HCC immunotherapy and targeted drug interventions remain poorly understood.

**Methods:**

RNA sequencing data and clinical characteristics of HCC patients were acquired from the TCGA database. To identify prognostically significant genes in HCC, we performed the limma package and conducted univariate Cox regression analysis. Subsequently, machine learning algorithms were employed to identify hub genes. To assess the immunological landscape of different HCC subtypes, we employed the CIBERSORT algorithm. Furthermore, single-cell RNA sequencing (scRNA-seq) was utilized to investigate the expression levels of ERFGs in immune cells and to explore intercellular communication within HCC tissues. The migratory capacity of HCC cells was evaluated using CCK-8 assays, while drug sensitivity prediction reliability was determined through wound-healing assays.

**Results:**

We have successfully identified a set of nine genes, termed EFRGs, that hold significant potential for the establishment of a hepatocellular carcinoma-specific prognostic model. Furthermore, leveraging the individual risk scores derived from this model, we were able to stratify patients into two distinct risk groups, unveiling notable disparities in terms of immune infiltration patterns and response to immunotherapy. Notably, the model’s capacity to accurately predict drug responses was substantiated through comprehensive experimental investigations, encompassing wound-healing assay, and CCK8 experiments conducted on the HepG2 and Huh7 cell lines.

**Conclusions:**

We constructed an EFRGs model that serves as valuable tools for prognostic assessment and decision-making support in the context of immunotherapy and chemotherapy.

## 1 Introduction

HCC stands as a prominent neoplasm within the realm of gastrointestinal malignancies afflicting adults (1, 2). Notably, the prognosis for HCC remains bleak. In cases where surgical resection is deemed unsuitable for advanced HCC patients, radiation therapy emerges as a strategic intervention to proficiently curb tumor advancement and mitigate symptomatic presentations, including postoperative radiotherapy. Nonetheless, the median survival rate in the aggregate remains suboptimal (3–6). Thus, there exists an imperative to discover efficacious biomarkers capable of prognosticating the outcomes of HCC patients, including their susceptibility to immunotherapy and chemotherapy. The identification of such biomarkers would furnish clinicians with invaluable guidance, facilitating the formulation of optimal treatment strategies.

Efferocytosis, an indispensable immune cell function, assumes a pivotal role in the elimination of aberrant cells, pathogens, and cellular debris (7–9). The term “efferocytosis” denotes the engulfment of one cell by another, commonly exemplifying the phagocytic uptake of apoptotic cells by macrophages (10, 11). This intricate process holds profound significance in upholding tissue homeostasis and averting the onset of inflammation. Efferocytosis constitutes a multifaceted process involving the recognition, phagocytic engulfment, and subsequent degradation of apoptotic cells by phagocytes. This intricate mechanism is meticulously regulated by a complex interplay of molecular signals and cellular receptors, serving to facilitate the prompt and efficient clearance of dying cells while mitigating the risk of autoimmune responses (12–14). Disruptions in the efferocytosis process have been implicated in a broad spectrum of pathological states, encompassing autoimmune disorders, chronic inflammatory conditions, and neoplastic diseases. Gaining a comprehensive understanding of the molecular mechanisms underlying efferocytosis presents substantial potential for the advancement of therapeutic interventions aimed at modulating immune responses and effectively addressing disorders associated with immune dysregulation.

Numerous cell lineages partake in the process of efferocytosis, each playing a role in the clearance of diverse cellular constituents within specific biological contexts. Neutrophils, for instance, possess the ability to release DNA fiber networks during inflammatory processes and are adept at engulfing both these networks and cellular remnants containing DNA (15, 16). Neutrophils, an indispensable entity within the immune system, exert cytotoxic effects on infected and cancerous cells, while also participating in efferocytosis for the clearance of deceased cells (17). Furthermore, dendritic cells and other immune cells actively contribute to the intricate process of efferocytosis (18, 19). Intriguingly, specific malignant tumor cells express receptors and ligands associated with efferocytosis, facilitating their engulfment of apoptotic cells within their microenvironment and aiding in immune evasion (20, 21). The multifaceted involvement of efferocytosis in tumor development exhibits a context-dependent role. During the initial stages of tumorigenesis, efferocytosis may exert a promotive influence on tumor growth by attenuating immune system attacks and facilitating the survival and proliferation of neoplastic cells through the clearance of apoptotic cells in their immediate vicinity (22). Additionally, tumor cells expressing receptors and ligands associated with efferocytosis can effectively evade immune responses, thereby fostering the progression of tumorigenesis (23–25). Nevertheless, during the late stages of tumor development, efferocytosis can switch its effect and inhibit tumor growth by promoting immune system recognition and attack against the tumor (26, 27). This dualistic function of efferocytosis in tumor biology underscores its complexity and highlights its potential as a therapeutic target for modulating immune responses and manipulating tumor progression. However, the precise implications of efferocytosis-associated genes in the progression and prognostication of hepatocellular carcinoma remain inadequately comprehended.

In this study, a comprehensive examination of HCC transcriptome dataset from the TCGA database was undertaken. By employing univariate and LASSO Cox regression analyses, we successfully ascertained nine distinctively expressed EFRGs, enabling the construction of a prognostic model for HCC. Remarkably, this model demonstrated significant predictive efficacy with regard to both patient prognosis and immune therapeutic response in the context of HCC. The expression profiles of EFRGs in immune cells were unveiled through scRNA-seq analysis. Furthermore, cellular experiments in the field of cell biology corroborated the potential of the EFRGs model as a predictive determinant of drug sensitivity. As a whole, this study constitutes the comprehensive bioinformatics exploration illuminating the crucial involvement of efferocytosis in HCC progression, encompassing aspects such as immune therapy response and prognostic risk prediction. These findings offer valuable insights to clinicians, aiding in the formulation of optimal treatment strategies.

## 2 Materials and methods

### 2.1 HCC data acquisition

In this investigation, we used the TCGA-LIHC cohort (374 LIHC and 50 normal tissue samples) on the TCGA data portal (https://portal.gdc.cancer.gov/) to acquire gene expression profiles and clinical data, such as TNM classification, age, gender, and overall survival (28). Additionally, we obtained the GSE14520 dataset from the GEO database (https://www.ncbi.nlm.nih.gov/geo/), which had 221 HCC samples, and the ICGC dataset (https://icgc.org/), which contained 240 HCC samples. For analysis, only data with comprehensive clinical information were used. Furthermore, single-cell data was sourced from the Tumor Immune Single-Cell Hub (TISCH2; http://tisch.comp-genomics.org), a comprehensive online repository of single-cell RNA-seq data that specifically focuses on tumor microenviroment (TME) (29, 30). Utilizing this resource, we systematically investigated TME heterogeneity across diverse datasets and cell types. The kmcellbank provided all of the HepG2 and Huh7 cell lines (KCB200507YJ and KCB200970YJ).

### 2.2 EFRGs resource

A comprehensive collection of 111 EFRGs was obtained from the GeneCards repository (31, 32).

### 2.3 Consensus clustering

For cluster analysis, we employed the “ConsensusClusterPlus” package and applied the k-means algorithm (33). To identify genes with significant alterations across distinct EFRGs clusters, differential expression analysis was conducted using the “limma” software. FDR < 0.05 and an absolute log2 fold change (|log2FC|) > 0.5 were used as criteria for determining the significance of changes.

### 2.4 Developing the EFRGs prognostic model

To discern prospective prognostic genes, we employed the LASSO regression analysis in TCGA-HCC cohort (34, 35), employing the “glmnet” package in the R programming language (36, 37). Through this methodology, we successfully identified a distinct ensemble of nine fundamental genes that constitute the foundation of a comprehensive risk signature (38). By employing the gene expression profiles of the aforementioned identified genes, we computed a personalized risk score for each patient within the studied cohort. Riskscore = e^(Exp. DYNLT1*0.176 + Exp.ADAM9*0.1235 + Exp.SCARB1*0.1192 + Exp.PPARG*0.1114 + Exp.HAVCR1*0.099 + Exp.GAPDH*0.0793 + Exp.LGALS3*0.0647 + Exp.SIRT6*0.0624 - Exp.IL33*0.0997). Subsequently, utilizing the median risk score as a threshold, the individuals diagnosed with HCC were categorized into distinct groups characterized as high-risk and low-risk. The performance of the model was evaluated through the utilization of a receiver operating characteristic (ROC) curve, which was generated employing the “timeROC” R package (39, 40). Furthermore, for model validation, we employed the GEO and IGCG cohorts. ROC curves were also generated using the “timeROC” R package, while the “survival” package facilitated the plotting of Kaplan-Meier survival curves to illustrate our findings (40).

### 2.5 TME estimation

To quantify the relative abundance of infiltrating immune cells, we employed CIBERSORT and ssGSEA R scripts. Utilizing CIBERSORT, we quantified the immune cell infiltration within each sample and performed intergroup comparisons (41). In order to investigate variations in the biological mechanisms linked to EFRGs, we performed Gene Set Variation Analysis (GSVA) using the gene set collection “c2.cp.kegg.v7.2.symbols.gmt” sourced from the MSigDB database (42, 43). ssGSEA method was performed to assess the level of immune cell infiltration (44). Furthermore, survival analysis was performed utilizing the “survival” and “survminer” packages in the R software.

### 2.6 Drug sensitivity prediction and validation

To identify ideal therapeutic options for individuals with HCC, we employed the “pRRophetic” R package to evaluate the half-maximal inhibitory concentration (IC50) values of diverse clinical medication (45). Subsequently, the cellular sensitivity to these drugs in HCC cells was assessed utilizing the CCK-8 assay, thereby evaluating their efficacy.

### 2.7 Tumor migration ability

HCC cells were plated in 6-well plates and cultivated until they achieved a confluency of 95%. To create a precise scratch in each well, a sterile 20-L plastic pipette tip was employed, followed by a gentle washing with PBS to remove any unattached cells and debris. The width of the scratch wounds was assessed at 0 and 36 hours by capturing photographs and subsequently measuring them using Image J software.

### 2.8 Statistical analysis

R 4.2.3 software was utilized for data processing, statistical analysis, and visualization purposes. The determination of the optimal cut-off value was achieved through the utilization of the “survminer” R package, while Kaplan-Meier analysis was carried out using the survival program. Comparisons between the two groups with respect to continuous variables were conducted utilizing the Wilcoxon-rank sum test, while the Spearman correlation analysis was employed to assess the interrelationships among continuous variables. Statistical significance was defined as *P* < 0.05 for all statistical analyses performed.

## 3 Results

### 3.1 Identification of EFRGs

**Figure 1:**
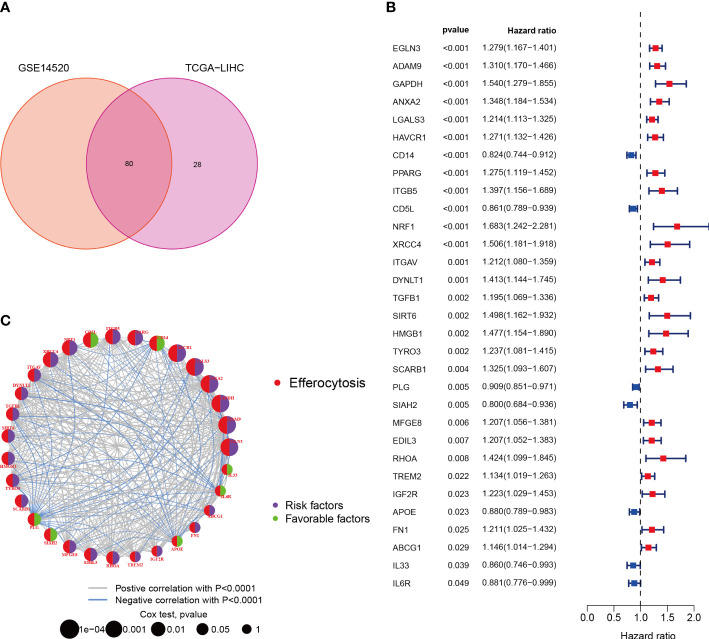
Characterization of Efferocytosis in HCC. **(A)** Identification of 80 genes associated with efferocytosis from hepatocellular carcinoma cohort. **(B)** 31 EFRGs associated with prognosis. **(C)** Network of 31 EFRGs.

**Figure 2:**
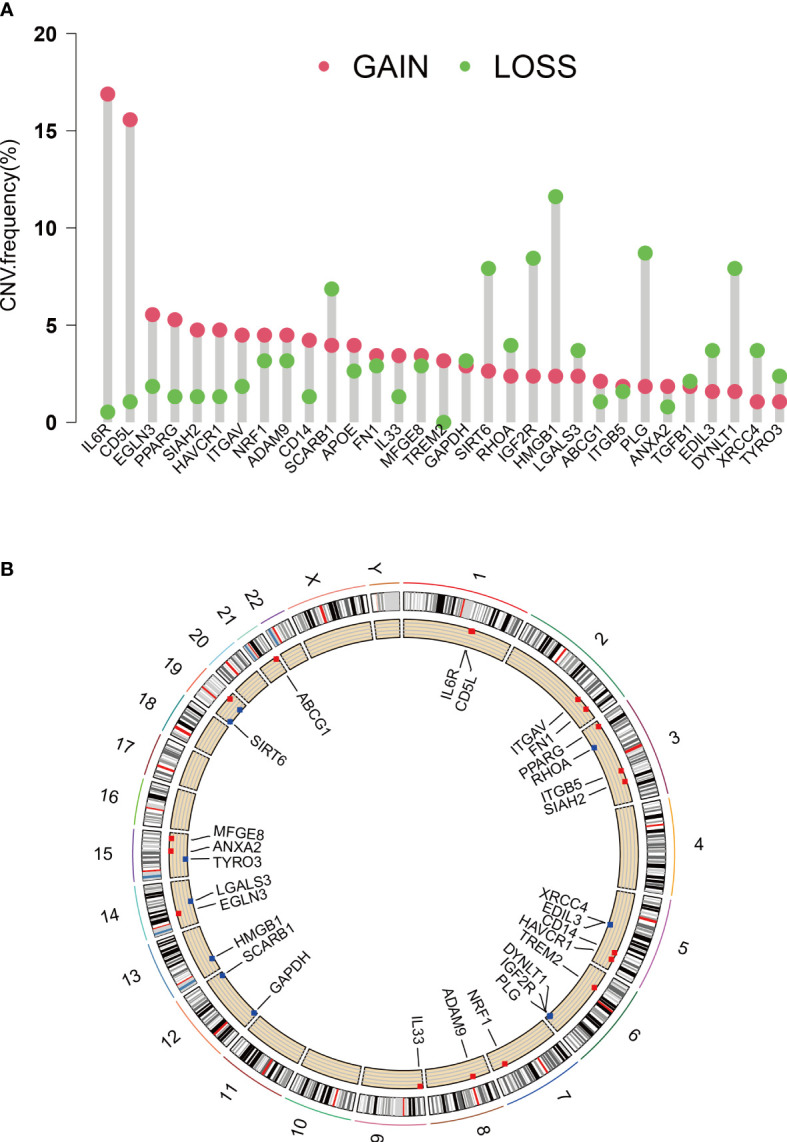
CNVs and chromosome region of EFRGs. **(A)** CNVs of 31 EFRGs. **(B)** Chromosome region of EFRGs.

EFRGs, a collection of 111 genes related to efferocytosis, were made available on the Genecards platform. The transcriptome data of 370 HCC tumor samples were obtained from the TCGA database. The GSE14520 cohort was accessed via the GEO website. Eighty out of the 111 EFRGs were found to be shared between two HCC cohorts, as depicted in a Venn diagram (**Figure 1A**). Subsequently, the “limma” R program was employed to discern 58 DEGs in HCC tumor and normal specimens. Among these 58 DEGs, 31 exhibited statistically significant associations with the prognosis of HCC patients as determined by univariate Cox regression analysis. Specifically, 24 DEGs were associated with a worse prognosis, while 7 DEGs were associated with a better prognosis (**Figure 1B**). To demonstrate the associations between the survival of HCC patients and the identified 31 EFRGs, as well as the interconnections among these EFRGs themselves, a network plot was employed (**Figure 1C**). Copy number variations (CNVs), which refer to variations in the number of DNA sequence copies in the genome that exhibit interindividual variability, play a significant role in neoplastic conditions and exhibit a robust correlation with tumor initiation, progression, and prognostication. Consequently, we conducted an in-depth investigation into the CNV alterations affecting the aforementioned 31 EFRGs in light of these established correlations. A prominent amplification of IL6R and CD5L is observed among the EFRGs, whereas a distinct copy number reduction is evident in HMGB1, PLG, DYNLT1, and IGF2R (**Figure 2A**). Furthermore, **Figure 2B** displays the genomic loci of these genes.

### 3.2 EFRGs subclusters identification

**Figure 3:**
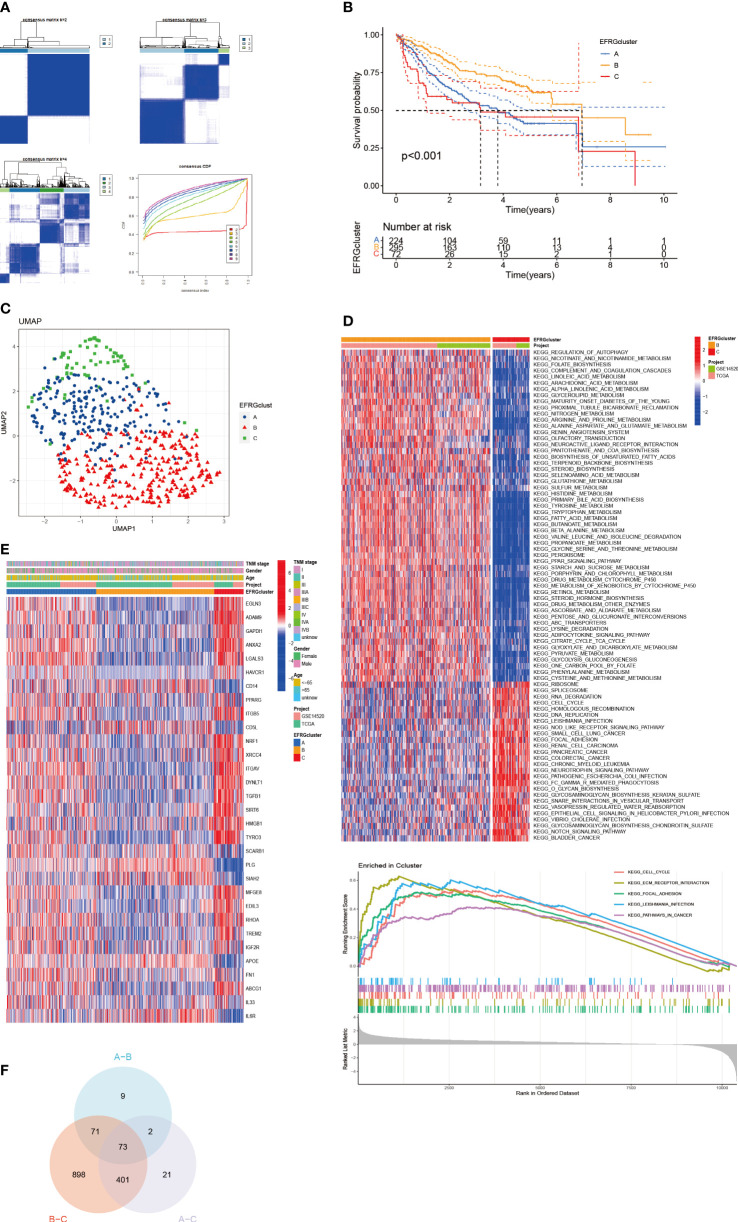
Subgroups of HCC differentiated by EFRGs. **(A)** The consensus clustering analysis yielded a satisfactory k = 3 consensus matrix, which was deemed acceptable. **(B)** Survival probabilities for three HCC subtypes were determined. **(C)** UMAP identified three distinct subtypes characterized by variations in the expression levels of EFRGs. **(D)** KEGG pathway enrichment. **(E)** Characteristics of 3 subtypes of EFRGs expression about clinical and pathological perspectives. **(F)** Clusters derived from differential expression of EFRGs exhibit overlapping regions as observed on Venn diagrams.

We used an integrative strategy using HCC samples from both the TCGA and GSE14520 cohorts in order to acquire thorough insights into the expression patterns of EFRGs implicated in carcinogenesis. By categorizing the data based on the expression patterns of 31 EFRGs, a reliable clustering technique was used to identify various subtypes within the HCC samples. K=3 was found to be the ideal number of clusters by analyzing the CDF (Cumulative Distribution Function) curve (**Figure 3A**). The integrated cohort was divided into three separate EFRG clusters as a result. After doing a survival study, it was shown that cluster B had considerably higher overall survival (OS) than cluster C (**Figure 3B**). The Uniform Manifold Approximation and Projection (UMAP) analysis revealed that the distribution of the three EFRG clusters was considerably different (**Figure 3C**). For each of the three subtypes, the expression of EFRGs was shown in a heat map along with the matching clinicopathological characteristics (**Figure 3E**). Given the stark differences between clusters B and C (**Figure 3D**), we used the GSVA software to explicitly analyze the differential enrichment of KEGG pathways. EFRGs are distributed differently throughout the three subtypes, identifying 73 genes that vary significantly between these subtypes (**Figure 3F**).

### 3.3 Immune infiltration in the EFRGs subtypes

**Figure 4:**
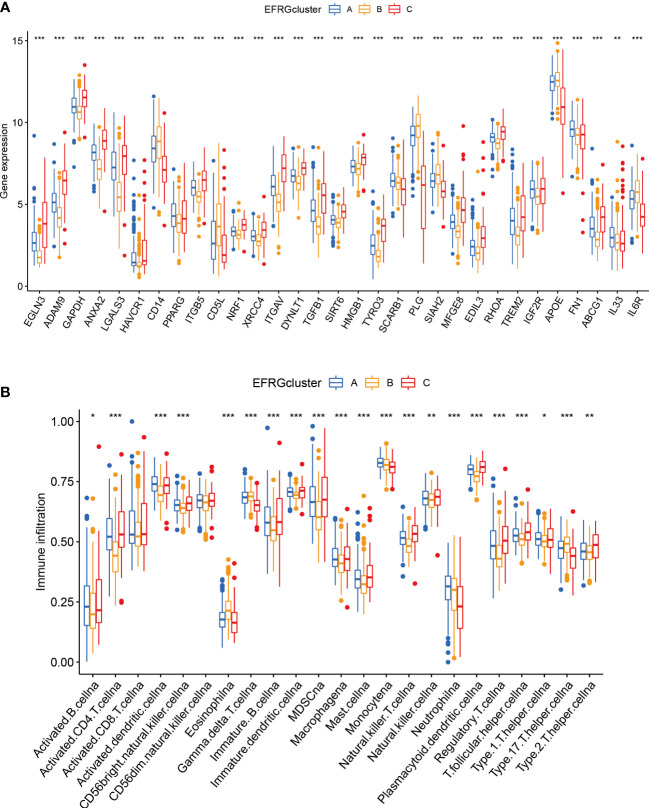
Immunity and gene expression patterns of EFRG subgroups. **(A)** 31 EFRGs expression profiles. **(B)** Patterns of immune infiltration across three HCC subtypes. *p < 0.05; **p < 0.01; ***p < 0.001.

The distribution of the EFRGs in the three subgroups was shown by visualizing their expression patterns. Notably, cluster A’s expression level for EFRGs was midway between clusters B and C, echoing the patterns of predictive survival time that had been noticed (**Figure 4A**). In terms of immune cell infiltration, cluster B showed a marked difference from the other two groups, with a much lower percentage of activated dendritic cells, NK cells, and macrophages (**Figure 4B**). This emphasizes how closely immune cells and efferocytosis are related in the prognostic subgroups of hepatocellular cancer.

### 3.4 Risk model construction and validation

**Figure 5:**
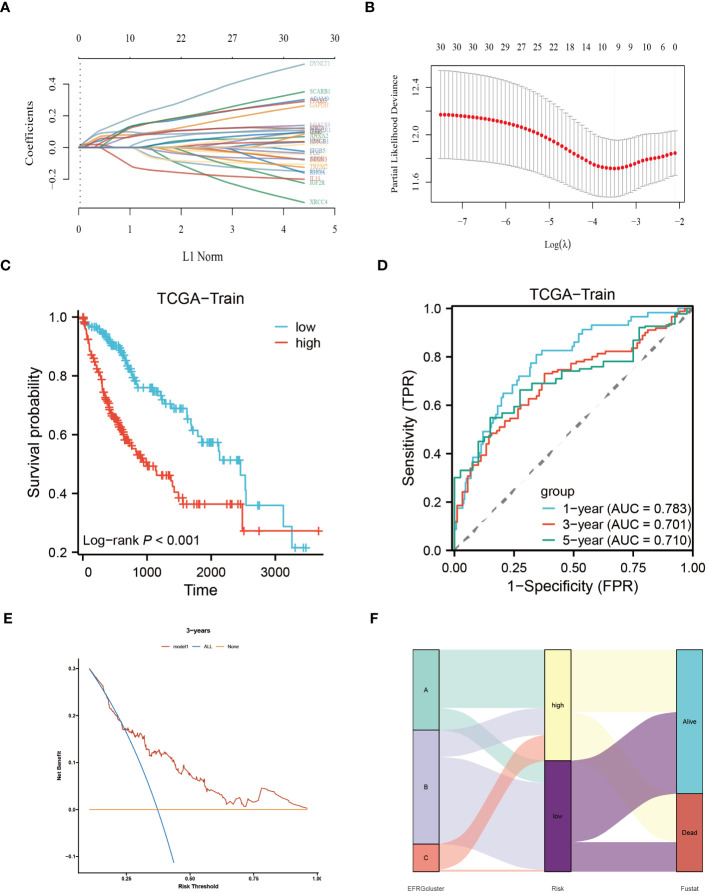
Identify the core EFRGs to build a prognostic model. **(A, B)** A total of 9 prognostic EFRGs were identified. **(C, D)** The TCGA cohort was employed to establish the training dataset for the prognostic model. **(E)** Decision curve analysis. **(F)** Diagram illustrating the clustering of EFRGs and their corresponding survival status through an alluvial representation.

**Figure 6:**
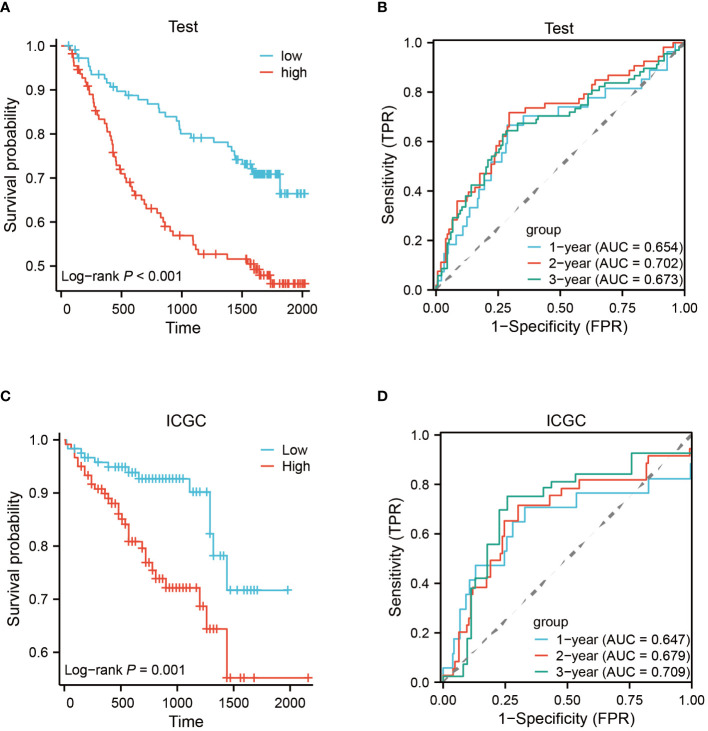
Testing and validation of prognostic EFRGs models. **(A, B)** Testing of EFRGs model. **(C, D)** Validation of EFRGs model.

To construct a rigorous HCC risk model, we conducted an analysis on 31 EFRGs using LASSO and multivariable Cox regression (**Figure 5A**). In the end, a total of 9 genes were used to calculate the HCC patients’ survival risk score (**Figure 5B**; **Supplementary Table S1**). All HCC patients had their risk scores calculated, and then they were split into high-risk and low-risk groups. The ROC curve illustrated the model’s ability to predict outcomes while the KM-plot was used to highlight the prognostic variations between two HCC subgroups with different risk (**Figures 5C, D**). Additionally, according to the decision curve, the EFRGs model may help HCC patients in the clinical setting as an auxiliary tool (**Figure 5E**). Cluster C of the EFRG subgroups was shown to have the worst prognosis by the Alluvial plot (**Figure 5F**). The good prognostic prediction power of the EFRGs model was further demonstrated by using the GEO and ICGC HCC cohorts as the testing and validation sets, respectively. The high-risk group had a shorter survival time (**Figures 6A, B**), and there were significant differences between them and the low-risk group (**Figures 6C, D**).

### 3.5 Immune infiltration landscape

**Figure 7:**
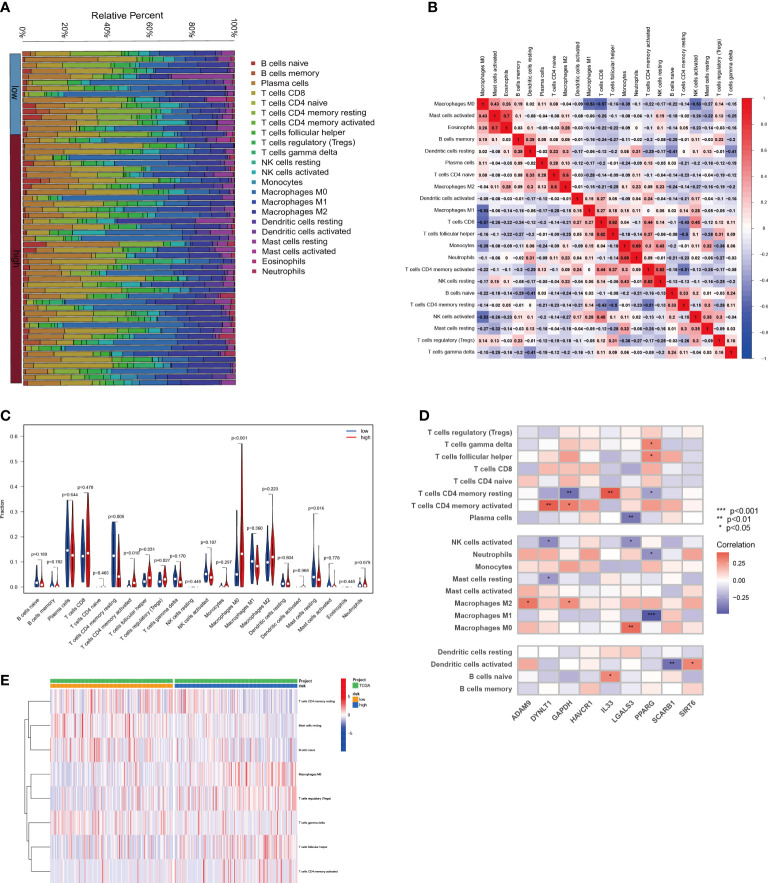
Relationship between immune infiltration and risk scores for HCC. **(A)** The proportion of immune cells responding in HCC patients with different risk scores. **(B)** The interrelationship between immune cells. **(C)** Differences in immune cell levels between different risk subgroups. **(D)** Correlation between immune cell populations and nine EFRGs. **(E)** Enrichment of immune cells in HCC patients with different risk scores.

The relationship between the tumor immunological microenvironment (TIM) and tumor development is well-established, with efferocytosis serving as a vital function for certain immune cells. To investigate potential variations in the immunological milieu among distinct subgroups of HCC classified based on efferocytosis, our study was designed. Employing the CIBERSORT R script, we assessed the relative proportions of diverse immune cell populations within each HCC sample (**Figure 7A**). Upon analyzing the intercellular associations of immune cells, a robust correlation was observed between active mast cells and eosinophils, followed by monocytes and neutrophils, activated CD4 memory T cells, and quiescent NK cells (**Figure 7B**). Notably, a pronounced disparity between the high-risk and low-risk groups in the infiltration of Macrophages M0 cells was identified (**Figures 7C, E**). The connections between nine EFRGs and several immune cells are illustrated (**Figure 7D**).

### 3.6 Immunotherapy response

**Figure 8:**
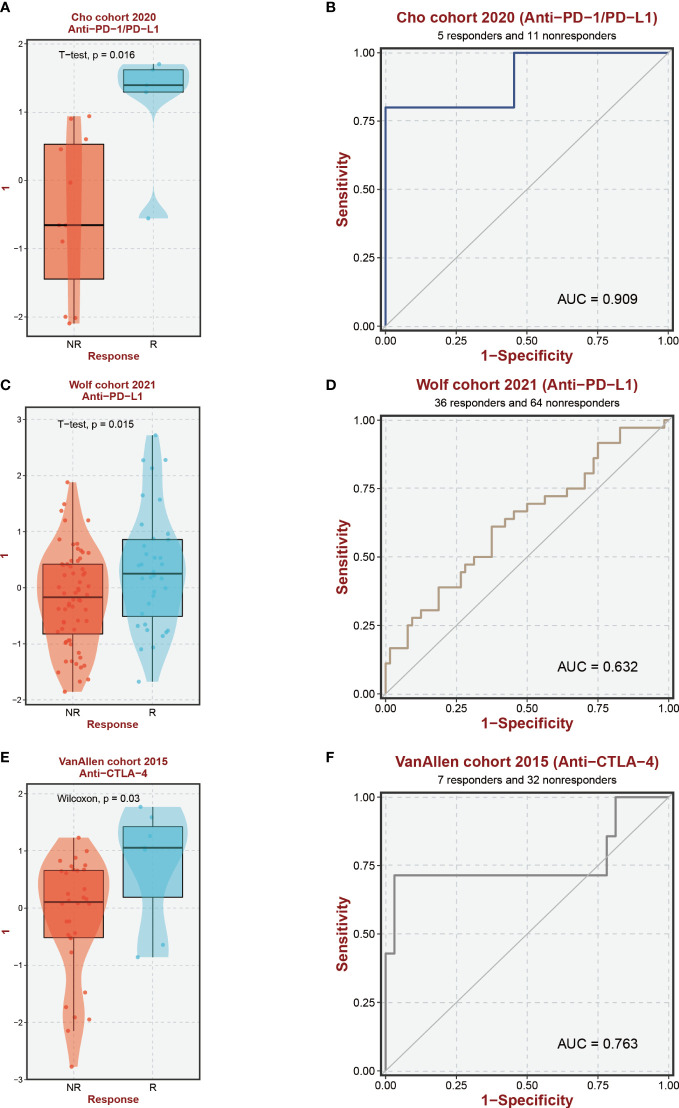
Immunotherapy response prediction. **(A, C, E)** Expression levels of EFRGs between different immune response groups. **(B, D, F)** Predictive efficacy of EFRGs on immunotherapy response.

Based on the findings from prior research, notable distinctions exist in the immunological microenvironments of high-risk and low-risk cohorts, characterized by variations in the infiltration levels of Macrophage M0, Tregs, and CD4 T cells. These modifications help to create an immunosuppressive milieu, which affects how differently the two groups respond to immunotherapy. Across three immune treatment cohorts, gratifyingly substantial variations in 9-EFRG expression levels between the responsive and non-responsive groups were observed (**Figures 8A, C, E**). Moreover, our EFRGs model exhibited remarkable precision in prognosticating the response to immune therapies targeting PD-1, PD-L1, and CTLA-4, showcasing exceptional predictive efficacy specifically for PD-1 response, as indicated by an area under the curve (AUC) surpassing 0.9 (**Figures 8B, D, F**).

### 3.7 Chemotherapy sensitivity prediction and validation

**Figure 9:**
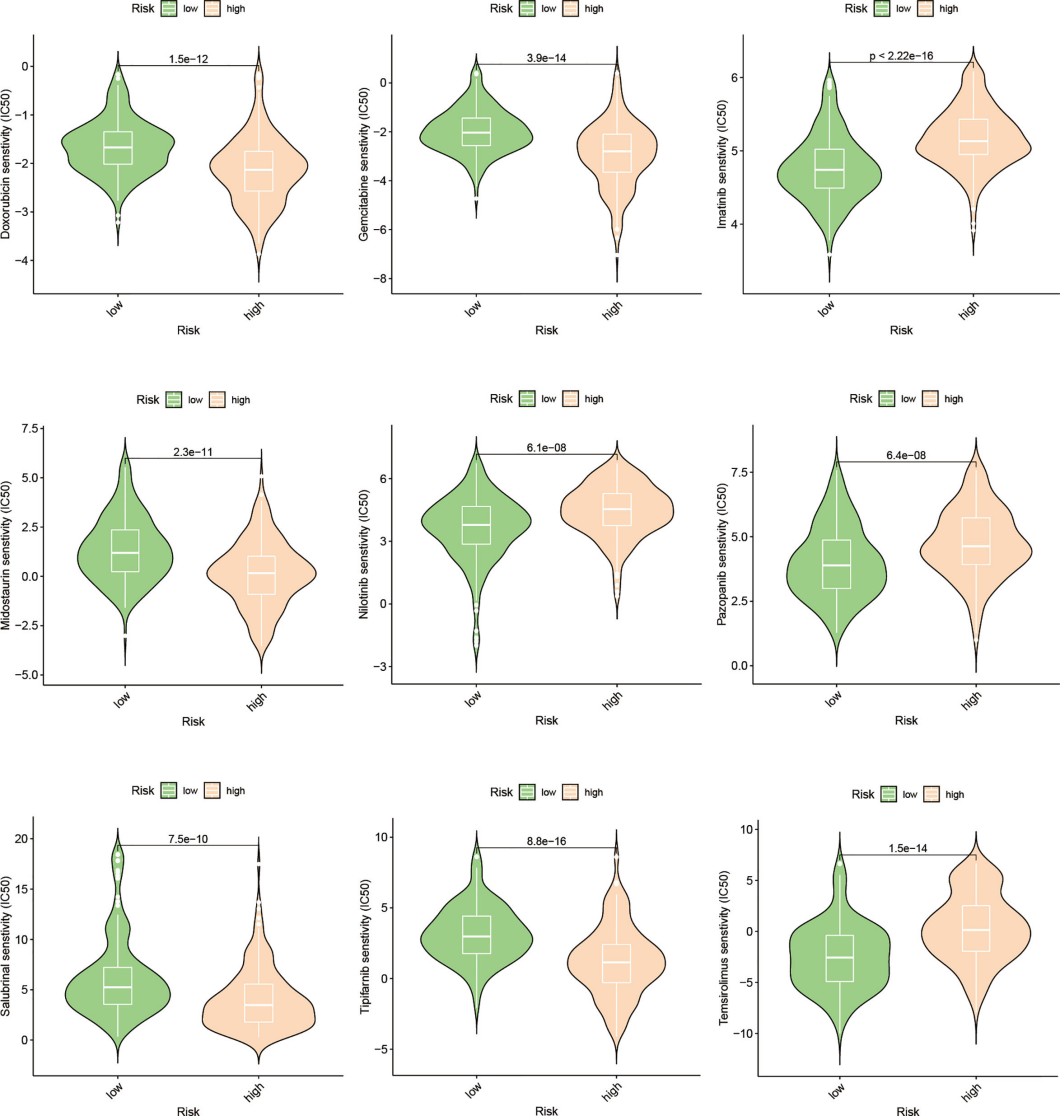
Drug prediction based on the expression pattern of EFRGs subgroups.

**Figure 10:**
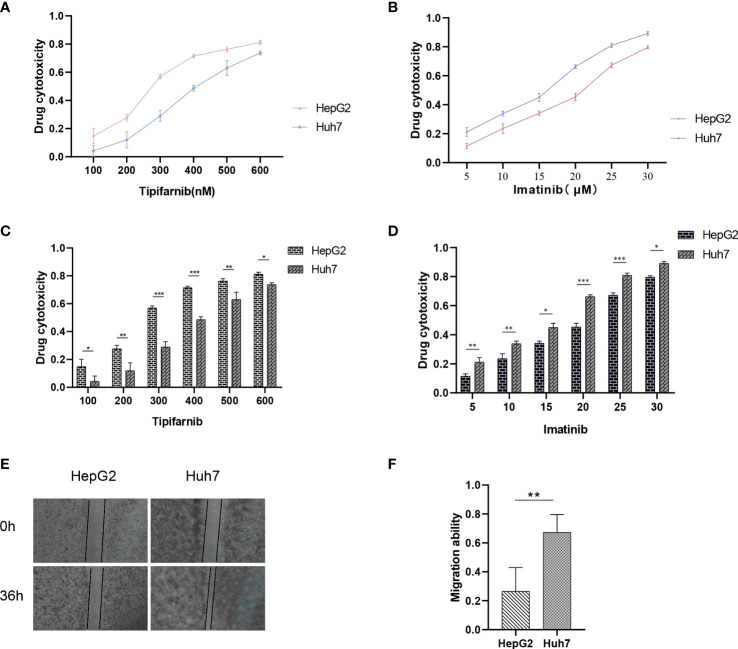
Drug sensitivity of HCC cell lines with different risk scores. **(A–D)** Drug sensitivity of Huh7 and HepG2 to different concentrations of Imatinib and Tipifarnib. **(E, F)** Migration ability between Huh7 and HepG2. *p < 0.05; **p < 0.01; ***p < 0.001.

Utilizing the “pRRophetic” R package, we conducted an evaluation to gauge the efficacy of chemotherapeutic agents in the treatment of HCC across various risk groups. Specifically, we employed this computational tool to compute the IC50 values for clinically utilized chemotherapeutic drugs in HCC treatment (**Figure 9**; **Supplementary Table S2**). In order to validate our findings, we conducted an assessment of risk scores among a diverse range of HCC cell lines. Subsequently, we selected the Huh7 and HepG2 cell lines to delineate distinct subgroups of HCC patients, representing those with high-risk and low-risk scores, respectively (**Figure 10**). Employing the CCK-8 assay, we observed disparate sensitivities of Huh7 and HepG2 cells towards etoposide, with the Huh7 cells, characterized by high-risk scores, demonstrating a greater degree of responsiveness compared to the HepG2 cells (**Figures 10B, D**). These observations are in accordance with the expected responses to drug sensitivity and offer additional validation for the prospective applicability of this chemotherapeutic agent as a precision therapeutic alternative for individuals with a heightened susceptibility to hepatocellular carcinoma.

### 3.8 Transwell and wound-healing assay

Previous analysis indicates that there are significant differences in the prognosis of HCC patients with different risks, which is closely related to tumor metastasis. Therefore, we aimed to assess the migration and invasion abilities between HCC cell lines of different risks. Huh7 and HepG2 cells were seeded in Transwell chambers, and the number of cells that crossed the chambers was observed at 24 hours and 48 hours, respectively (**Figures 10A, C**). Additionally, a wound-healing assay was performed to provide a more intuitive reflection of the differences in migration abilities between Huh7 and HepG2 cells, which represent different risk levels (**Figures 10E, F**). This explains why our model can accurately reflect the differences in prognosis among HCC patients with different risks.

### 3.9 Single-cell transcriptome analysis

**Figure 11:**
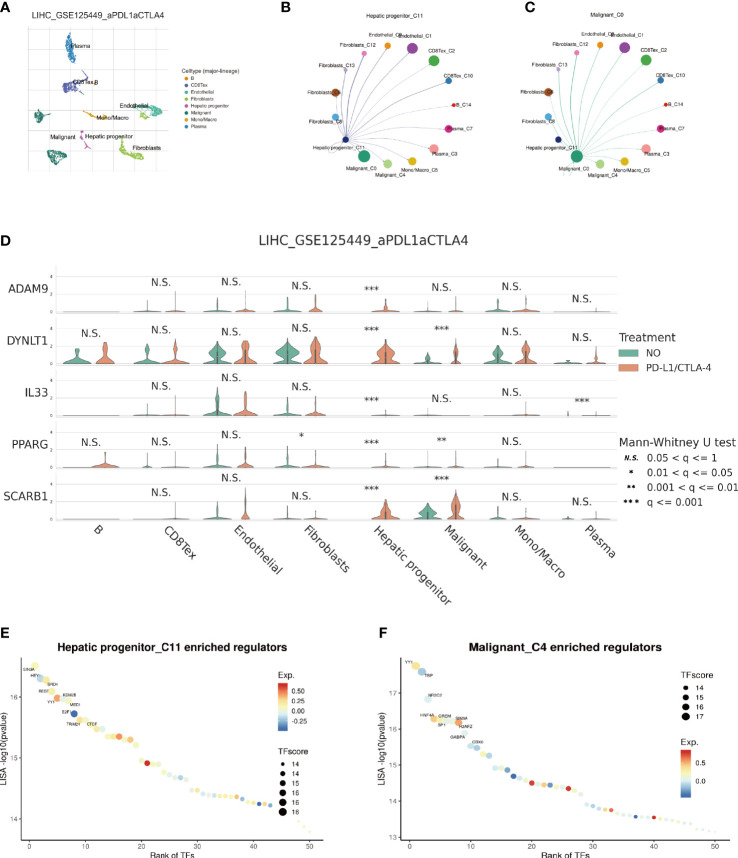
Single-cell sequencing analysis of immunotherapy in HCC patients. **(A)** Cells are divided into 8 clusters. **(B, C)** Cellular Communication. **(D)** Expression Changes of EFRGs in 8 cells clusters. **(E, F)** Identification of key transcriptional regulators.

The emergence of single-cell technology has greatly enhanced our comprehensive understanding of cellular populations as a whole. In the following investigation, we explore the expression levels of EFRGs in diverse cell types and their intercellular communication. To gain deeper insights into the changes in EFRG expression levels across distinct cell populations within HCC patients after PD-L1 and CTLA-4 immunotherapy, we selected the LIHC_GSE125449 aPDLaCTLA4 cohort for single-cell analysis (**Figure 11A**). Given the variation in the sensitivity of tumor patients to immunotherapy, we compared the expression levels of EFRGs between HCC patients who received PD-L1/CTLA-4 treatment and those who did not (**Figure 11C**). Furthermore, the connections between tumor cells, hepatic progenitors, and various immune cells were elucidated. Significant associations were observed among HCC cells, hepatic progenitors, and CD8 T cells (**Figures 11B, C**). The identification of transcriptional regulators (TFs) that govern differential expression is crucial for understanding the underlying gene regulatory networks. Therefore, we predicted the TFs that shape the expression patterns in different scRNA-seq clusters. SIN3A and YY1 were identified as key regulators in hepatic progenitors and HCC cells (**Figures 11E, F**).

## 4 Discussion

The management of HCC, an exceedingly aggressive and metastatic cancer characterized by a high rate of recurrence, poses substantial challenges in clinical care. HCC has an astonishingly poor 5-year survival rate, underscoring the urgent need for better treatment strategies (1, 46). Despite the progress made in diverse therapeutic approaches, including chemotherapy and immunotherapy, the intrinsic heterogeneity of HCC tumors and their associated adverse prognostic outcomes continue to pose significant challenges (47, 48). Consequently, the identification of prognostic markers and biomarkers holds paramount importance in accurately evaluating treatment response (49–52), thereby facilitating enhanced clinical decision-making for individuals suffering from HCC.

Efferocytosis, a pivotal immune system mechanism involved in the clearance of apoptotic cells to preserve tissue homeostasis, exerts a substantial impact on a wide range of physiological and pathological processes. This includes its influence on tumor development and progression (7–9). Neoplastic cells in the tumor microenvironment of HCC go through repeated cycles of proliferation and death, releasing a variety of inflammatory mediators and triggering subsequent inflammatory responses. These inflammatory reactions encourage the attraction and stimulation of immune cells with efferocytic properties (12–14). Surprisingly, new research has shown that cancerous cells can use the efferocytosis mechanism to circumvent immune identification and immune monitorin (53). Tumor cells are able to display molecular signals associated with efferocytosis on their cellular membrane and evade the immune system by modifying the polarization and number of macrophages (54–56). There is currently a dearth of study on efferocytosis, particularly in relation to the choice of immune treatment and malignant prognosis. With the use of multi-omics research, our work intends to clarify the role of efferocytosis in the development and therapy of HCC.

Using a clinical approach, we first screened a cohort of HCC patients to find 80 common EFRGs (**Figure 1A**). Univariate regression analysis was then performed to find 31 EFRGs that were substantially linked with the prognosis of HCC (**Figure 1B**). We divided HCC patients into three categories based on the levels of EFRG expression because we believed that efferocytosis played a crucial role in tumor development (**Figure 3B**). These subgroups showed substantial variations in overall survival. This shows that efferocytosis-related gene expression levels have a considerable impact on the prognosis of HCC patients, potentially acting as prognostic biomarkers. We further clarified the relationships between these EFRGs and clinical characteristics (**Figure 3E**). Lasso analysis was used to analyze the connection between the expression patterns of 31 EFRGs and survival in order to learn more about the function of EFRGs in hepatocellular carcinoma prognosis. As a result, we created a nine-EFRG prognostic prediction model (**Figures 5A, B**). The model’s training data came from the TCGA cohort, while its testing and validation data came from the GSE14520 and ICGC cohorts, respectively. The prognostic prediction capacity of our EFRGs model was excellent overall (**Figures 6B, D**). In the course of a survival study, it was shown that patients with high-risk and low-risk HCC had significantly different survival rates (*P* < 0.001) (**Figures 6A, B**). The model could improve clinical patients’ chances of survival, according to DCA (**Figure 5E**).

Immune cells play critical roles in the development of diseases (57–59). By engulfing apoptotic cells during the process of efferocytosis, immune cells serve a critical role in controlling the growth of tumors (7–9). Effective efferocytosis makes it easier to remove apoptotic cells, which prevents inflammatory responses and slows the growth of tumors (22). However, immune cells with abnormal functions may have impaired efferocytosis, which would therefore encourage the formation of tumors and immune evasion. Because there were substantial variations in immune cell infiltration across the three subgroups of HCC, we further evaluated the levels of immune cell infiltration, including dendritic cells, CD4 T cells, and macrophages (**Figure 4B**). We further examined the immune landscape infiltration in high-risk and low-risk HCC patients using the EFRGs predictive model (**Figure 7**). Patients with high-risk HCC showed concentration of Macrophages M0, regulatory T cells (Tregs), follicular helper T cells, and activated CD4 memory T cells. Meanwhile, naïve B cells and resting Mast cells were enriched in low-risk HCC patients (**Figure 7E**). Interestingly, activated Mast cells and memory B cells showed a significant connection with macrophages M0 (**Figure 7B**). In patients with high-risk HCC, we found that the amount of Macrophages M0 infiltration was considerably greater (**Figure 7C**). So we examined the relationship between nine EFRGs and immune cell infiltration (**Figure 7D**). The findings showed that LGALS3 had the strongest connection with Macrophages M0, indicating that LGALS3 could be an important EFRG that affects how well macrophages perform efferocytosis.

In-depth research has been done on immunotherapy, which has emerged as a crucial therapeutic approach in the management of cancer (60–62). This treatment strategy makes use of the immune system’s innate capacity to identify and get rid of cancerous cells (63–65). Macrophages and the crucial molecules CTLA-4, PD-1, and PD-L1 have a complex relationship (66–69). In order to assess the potential impact of disparate levels of macrophage infiltration on the response to immune therapy, we investigated whether such variation could influence the therapeutic efficacy (70). Our findings revealed significant differences in the expression of nine EFRGs between the groups responsive and non-responsive to immune therapy (**Figures 8A, C, E**). Consequently, our prognostic model exhibits the ability to predict the response of HCC to PD-1, PD-L1, and CTLA-4 antibodies (**Figures 8B, D, F**). Single-cell analysis provides a profound understanding of the expression levels of genes among different cells within the same sample, offering guidance in the identification of critical cellular subtypes that exert fundamental functions. We were intrigued by the impact of the immune checkpoint inhibitors PDL1/CTLA-4 on the tumor microenvironment of HCC. Therefore, employing single-cell analysis, we compared the expression level variations of EFRGs among various cellular components within the immune microenvironment between samples treated with anti-PDL1/CTLA-4 and control HCC samples (**Figure 11A**). Hepatic progenitor cells and HCC cells were identified as the cell populations exhibiting the most significant changes in EFRG expression levels (**Figure 11D**). Given that high EFRG expression is indicative of improved response to immune therapy (**Figure 8**), these two cell types may serve as crucial target cells mediating the anti-tumor effects of anti-PDL1/CTLA-4.

Presently, the investigation into the contribution of efferocytosis to tumor drug resistance remains limited. Conversely, considerable research has been dedicated to studying the involvement of immune cells in tumor drug resistance. Considering the intimate relationship between efferocytosis and immune cells, we aimed to explore whether efferocytosis could serve as a predictive factor for the sensitivity of HCC to chemotherapy agents. To this end, we leveraged the “pRRophetic” R package to analyze and compare the IC50 values of distinct drugs between high-risk and low-risk cohorts, with the intention of identifying significant difference. Significant disparities in drug sensitivity were observed among high-risk and low-risk HCC patients when assessing the response to 25 chemotherapy drugs (**Figure 9**). Notably, high-risk HCC patients exhibited heightened sensitivity to Imatinib in comparison to their low-risk counterparts. Furthermore, CCK-8 assays substantiated that, under equimolar drug concentrations, high-risk Huh7 cells displayed greater susceptibility to Imatinib than HepG2 cells (*P* < 0.05) (**Figures 10B, D**). The obtained results suggest that our prognostic model based on EFRGs can potentially offer insights into the responsiveness of HCC to chemotherapy agents, thereby offering valuable guidance for clinical drug selection in patient management.

Despite providing numerous valuable findings, our study has certain limitations that need to be acknowledged. Firstly, this research heavily relies on publicly available datasets and is solely based on three HCC datasets, which may introduce selection bias. Therefore, further validation of the EFRGs prognostic model in HCC patients is warranted using larger clinical cohorts to enhance the credibility of the results. Additionally, the elucidation of the immune landscape requires validation through *in vivo* and *in vitro* experiments. Further investigations into the mechanisms underlying the impact of efferocytosis on HCC progression need to be elucidated through a series of cellular experiments. Nevertheless, it is worth emphasizing that our findings regarding efferocytosis highlight the significant value of EFRGs, establishing a connection between efferocytosis, the immune microenvironment, and the prognosis of HCC patients. Furthermore, the predictive value of our EFRGs model in prognosis, immune therapy, and chemotherapy has been confirmed. These findings hold promising potential for offering new directions in the clinical treatment of HCC.

## 5 Conclusion

Tumor growth is significantly influenced by efferocytosis, which plays a crucial role in physiological balance and disease pathogenesis. In this work, we developed a prognostic prediction model specifically for HCC and created a unique gene signature made up of 9 efferocytosis-related genes. The use of this approach shows potential for aiding in the development of tailored treatment plans for those with HCC. Additionally, the discovery of a link between immune microenvironment genes and the process of efferocytosis has opened up a brand-new field for the development of immunotherapeutic approaches. The effectiveness of anticancer therapy in HCC can be increased, increasing the susceptibility of HCC to treatment, by focusing on crucial efferocytosis-related genes.

## Data availability statement

The original contributions presented in the study are included in the article/**Supplementary Material**. Further inquiries can be directed to the corresponding author.

## Author contributions

Conceptualization, CyL. Writing original draft preparation, TL. Visualization, TL and CL. Data resources, JZ and HH. Supervision, validation, and funding acquisition, CyL. Review and editing, CyL. All authors reviewed and approved the final manuscript.
